# Statistical analysis considerations within longitudinal studies of physical qualities in youth athletes: A qualitative systematic methodological review

**DOI:** 10.1371/journal.pone.0270336

**Published:** 2022-07-07

**Authors:** Cameron Owen, Kevin Till, Josh Darrall-Jones, Ben Jones

**Affiliations:** 1 Carnegie Applied Rugby Research (CARR) Centre, Leeds Beckett University, Carnegie School of Sport, Leeds, United Kingdom; 2 England Performance Unit, The Rugby Football League, Leeds, United Kingdom; 3 British Swimming, Loughborough, United Kingdom; 4 Leeds Rhinos Rugby League Club, Leeds, United Kingdom; 5 School of Science and Technology, University of New England, Armidale, NSW, Australia; 6 Department of Human Biology, Division of Exercise Science and Sports Medicine, Faculty of Health Sciences, The University of Cape Town and the Sports Science Institute of South Africa, Cape Town, South Africa; Nottingham Trent University, UNITED KINGDOM

## Abstract

**Background:**

The evaluation of physical qualities in talent identification and development systems is vital and commonplace in supporting youth athletes towards elite sport. However, the complex and dynamic development of physical qualities in addition to temporal challenges associated with the research design, such as unstructured data collection and missing data, requires appropriate statistical methods to be applied in research to optimise the understanding and knowledge of long-term physical development.

**Aim:**

To collate and evaluate the application of methodological and statistical methods used in studies investigating the development of physical qualities within youth athletes.

**Methods:**

Electronic databases were systematically searched form the earliest record to June 2021 and reference lists were hand searched in accordance with the PRISMA guidelines. Studies were included if they tested physical qualities over a minimum of 3 timepoints, were observational in nature and used youth sporting populations.

**Results:**

Forty articles met the inclusion criteria. The statistical analysis methods applied were qualitatively assessed against the theoretical underpinnings (i.e. multidimensional development, non-linear change and between and within athlete change) and temporal challenges (i.e. time variant and invariant variables, missing data, treatment of time and repeated measures) encountered with longitudinal physical testing research. Multilevel models were implemented most frequently (50%) and the most appropriately used statistical analysis method when qualitatively compared against the longitudinal challenges. Independent groups ANOVA, MANOVA and X^2^ were also used, yet failed to address any of the challenges posed within longitudinal physical testing research.

**Conclusions:**

This methodological review identified the statistical methods currently employed within longitudinal physical testing research and addressed the theoretical and temporal challenges faced in longitudinal physical testing research with varying success. The findings can be used to support the selection of statistical methods when evaluating the development of youth athletes through the consideration of the challenges presented.

## Introduction

National governing bodies employ talent identification and development systems to support athletes with potential to excel in elite sport [1–3]. Whilst the evaluation of talented athletes within such systems is complex, the assessment and development of physical qualities is common in all sports due to their relationship with enhanced sport performance [4], reduced injury risk [5, 6] and future career attainment [7–12]. However, like talent, the development of physical qualities in youth athletes is dynamic, non-linear and confounded by multiple factors (e.g. chronological age, biological maturation, training age/status) complicating the understanding of their development [3, 13]. It is this complexity that differentiates the development of physical qualities in youth and senior athletes, as the effects of developmental and biological factors are not observed within senior athletes and therefore require different considerations within the analyses when quantifying change over time [2]. Although such research is required to support the knowledge and understanding of the long-term physical development of youth athletes there are several challenges associated with the research design and the subsequent application of statistical analyses in this area.

Research quantifying the physical qualities of athletes often utilises cross-sectional ‘one-off’ assessments, comparing between age grades (e.g., Under 13 vs. Under 14) to demonstrate the development and change in qualities over time [1]. However, assumptions regarding the within athlete development of physical qualities are based on the general principles observed between individual athletes, rather than a true change over time [14, 15]. For example, the difference in body mass or muscular strength between two age groups is not equivalent to the within athlete development. As a result, such designs are limited in their ability to accommodate for the effect of between athlete differences (e.g., chronological age, training age, maturation) on physical development, thus failing to address theoretical underpinnings by disregarding inter-athlete variability and consequently their implications for talent identification and development. In comparison, longitudinal research using repeated observations allows for the inclusion of particular exposures (e.g., differences in training load or training age) and the correction of cohort effects (e.g. chronological age and maturation) to allow examination of the within athlete change over time and the factors that influence this rate of change [16]. Whilst longitudinal methodology increases the confidence in any inferences made regarding the change or causality of observations [17], it is not without its limitations. In addition to being both time and financially expensive, longitudinal research requires the consideration of temporal design issues such as dealing with dependencies created by repeated measures (i.e. non-independence of data collected from a single athlete), missing and unbalanced data, separating group and individual athlete change, time-varying, -invariant and -related covariates and specifying the role of time/temporality [14]. Therefore, to optimise the use of longitudinal study designs it is important for researchers to consider such issues during the selection of appropriate statistical analyses to effectively answer the underpinning research questions and translate research findings into practice.

The discussion surrounding the application of statistical methods used within physical testing is currently limited. Park and Schutz [18] summarise the benefits of using latent growth models to analyse longitudinal physical testing data, highlighting their ability to identify individual and group levels of change, specify non-linear development, accommodate uneven spacing of date collection, account for measurement error, allow for multiple predictors of change and provide flexibility to develop the model. Although an overview of the application of latent growth models is provided, there is a lack of consideration of the benefits of other methods and their strengths such as hierarchical modelling and unbalanced study designs [19]. Support for the selection of analysis methods may also be supported from other areas of sports science. For example, Hamaker and Muthén [20] address the issues of separating within and between individual slopes from the group level of change within sports psychology. By providing an example where individual growth trajectories may not resemble the slope identified for the whole sample, (i.e. the within and between slopes are different), the importance of centring techniques in multilevel modelling and structured equation models are identified. The method presented by Hamaker and Muthén [20] is based on a single outcome and predictor relationship and the application within multivariable or multivariate approaches is not discussed, potentially limiting the application to understanding the complex development of physical qualities. An evaluation of the current statistical practices employed within physical testing will therefore provide further information on the most appropriate methods to address the specific challenges faced.

Systematic reviews are effective methods to summarise and synthesise the current research literature but fail to address the appropriateness of the statistical analysis methods employed [21]. Methodological reviews, although not used extensively, can provide a useful alternative to evaluate the statistical analysis methods found within the current literature and inform future research [22]. For example, Windt et al. [23] comprehensively evaluated the statistical analysis methods used to assess the workload-injury relationship in team sport athletes using Collins threefold alignment [15]. According to Collins [15], theoretical underpinnings (i.e. the characteristics of change over time such as the shape of change and function of variables on change) and temporal design (i.e. timing, frequency and spacing of observations) should be used to guide the selection of appropriate statistical analyses to maximise the utility of the data collected. Windt et al. [23] demonstrated that multilevel modelling and frailty models were more appropriate in the context of workload injury relationships due to their ability to address the multifactorial aetiology, between- and within-athlete differences and include both time varying and time invariant variables. However, these findings should not be generalised across all research studies, especially physical testing where the challenges faced in collecting data and the underpinning theory are different to the workload injury relationship. For example, while the development of physical qualities in youth athletes are multidimensional and dynamic, and therefore similar to workload-injury aetiology, the dependent variable is continuous in nature compared to the binary outcomes identified in injury research. Differences in the temporal demands of data collection are also apparent as workload-injury data is considered intensive longitudinal data (>20 observations) due to its frequent data collection to capture irregular fluctuations, whereas physical quality assessments are longitudinal panel data (<8 observations) due to the less frequent collection in order to observe long-term change and not the regular fluctuations (i.e., fatigue monitoring). Consequently, data collection is time unstructured (collected at irregular timepoints) and greater participant dropout could be observed over the duration of a study. It is therefore important that when analysing physical testing data longitudinally, the unique theoretical underpinnings and temporal challenges faced are considered when analysis techniques are selected by researchers.

With the increasing call for the implementation of national physical testing batteries and need for longitudinal research within youth sport [24, 25] it seems appropriate to consider the current longitudinal methods applied within research, alongside the statistical analyses employed, to increase the efficacy of findings within large datasets. Therefore, the aims of this qualitative systematic methodological review were to 1) identify the methodological and statistical analyses used within the youth athlete physical development literature and 2) evaluate the degree to which the statistical analyses applied address the underpinning theory and design of physical testing data collection. These findings can be used by researchers when selecting appropriate statistical analysis techniques for analysing physical testing data to optimise its usefulness and enhance the understanding of physical development and as such inform athlete development practices.

## Methods

### Search strategy

A systematic search of online databases (PubMed, MedLine, Scopus, CINAHL and SportDiscuss) was performed identifying papers from the earliest record to June 2021. This search was conducted according to the Preferred Reporting Items for Systematic Reviews and Metanalyses (PRISMA) [26]. The PRISMA checklist is reported in S1 File. The review was not registered and the protocol was not published prior to its commencement. Key words were used to identify appropriate literature relating to the data type, age, participation level and physical qualities linked using Boolean terms (Table 1). Reference lists were also manually searched for any further articles.

**Table 1:** Search terms used for systematic search of databases cased on data type, age, participation level and testing.

Data type	Age	Participation level	Testing
Longitudinal	academy OR youth OR adolescent OR junior	talent OR pathway OR elite OR academy OR club NOT education	‘Fitness testing’ OR ‘physical characteristics’ OR ‘physical qualities’ OR ‘physical performance’ OR ‘physical profile’ OR anthropometric OR ‘body height’ OR ‘body weight’ OR skinfold OR ‘body composition’ OR ‘body fat’ OR power OR ‘countermovement jump’ OR ‘vertical jump’ OR ‘muscular strength’ OR acceleration OR speed OR sprint OR running OR agility OR ‘change of direction’ OR fitness OR ‘physical fitness’ OR ‘aerobic capacity’ OR ‘cardiorespiratory fitness’ OR ‘repeated-sprint ability’ OR ‘anaerobic’

### Study selection

Following the removal of duplicates, two reviewers (CO, JDJ) screened the titles and abstracts for the eligibility criteria. Any disagreements were resolved by a discussion between the reviewers. For the remaining articles full texts were screened against the inclusion criteria, with the authors not blinded to the reviewers.

A study was included if it assessed the longitudinal development of physical qualities in youth athletes (aged under 21 years). The definition of longitudinal research was a minimum of three timepoints (mixed-longitudinal designs where the observation range included fewer timepoints were also accepted) due to the limitations of only capturing two observations such as the inability to identify non-linear relationships and account for measurement error [27]. Furthermore, studies were only included if they were observational in nature and intervention studies were excluded. Studies from youth sporting populations were accepted, for example clubs, academies or talent systems. If an article was not in English it was included on the provision an English version could be acquired. Only articles from peer reviewed journals were considered. Book chapters, abstracts and pre-prints were excluded.

### Extraction

For all articles, information regarding the publication (author and publication year), study length and population (sport, level, number of participants and timepoints) were extracted. Details on the statistical analysis method selected (i.e., method, justification for use and checking of assumptions and model fit), and dependent and independent variables used within each article were also noted. Finally, additional information relating to the theoretical underpinnings (multidimensional analysis, identification of non-linear change and group and individual athlete change) and temporal factors (time varying and invariant variables, missing and unbalanced data, how time is treated within the model and the dependency created by repeated measures) relating to physical testing were also obtained. All information was collated in Microsoft Excel (Microsoft Corporation, Washington, United States of America) where methodological and statistical information could be counted and summarised for reporting.

## Results

### Identification and selection of studies

**Fig 1:**
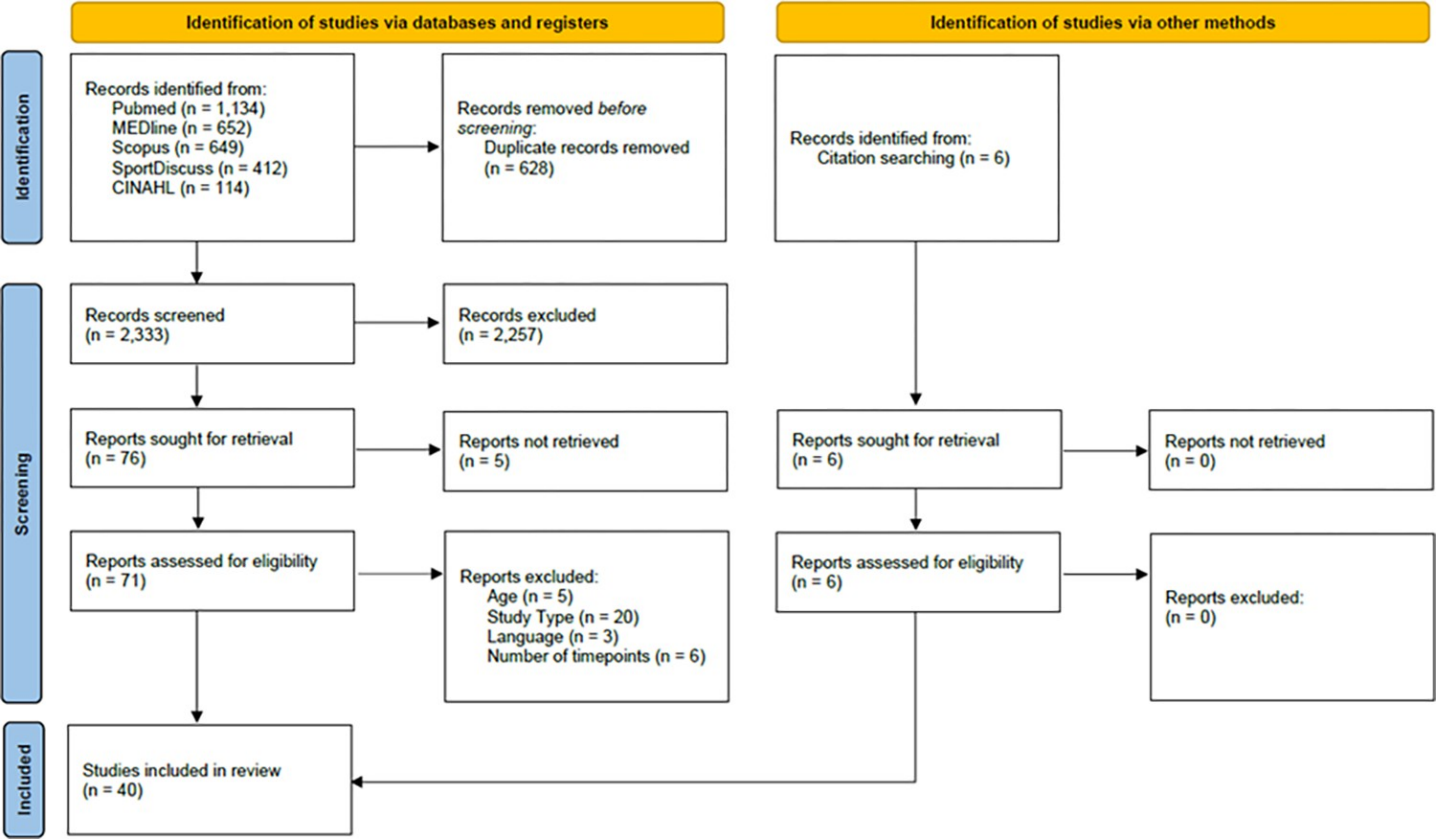
Flow of selection process of eligible studies for qualitative synthesis.

A total of 40 studies were identified for inclusion within this systematic methodological review (Fig 1). The search initially identified 2,961 articles with 628 duplicates removed. Of the remaining 2,333 articles, 2,257 were excluded following the first stage of screening. Five articles were not retrieved, with a further 34 articles removed following a review of the full text. This resulted in 34 studies being eligible with a further six included following a hand search of the reference lists.

### Study characteristics

Table 2 shows the characteristics of the studies which met the inclusion criteria. These articles were published between 1992 to 2020. A range of sports were assessed with the most common including soccer (n = 18; 45%) and rugby league (n = 9; 23%). Field hockey and tennis were each included in two studies, while rugby union, skiing, paddling, badminton, handball, basketball, sprint and mixed (swimming and racket sports) featured in one. Participants were recruited from academies (n = 12; 30%), clubs (n = 5; 13%), talent development programmes (n = 4; 10%), talent identification programmes, national level athletes, professional/elite clubs and performance pathways (n = 3; 8%), mixed level (n = 2; 5%), elite schools, centre of excellences or were top 10 national athletes (n = 1; 3%). The mean number of participants across the included studies was 264, median 81, and range 7 to 2,875. Studies were completed over a mean period of 4 years/seasons, with a median of 4 years and range of 1 to 11 years. Studies more frequently used male participants (n = 32; 80%), with mixed (n = 5; 13%) and female (n = 2; 5%) cohorts more infrequent.

**Table 2:** Summary of articles that investigated longitudinal fitness testing data.

Author	Sample size	Sex	Age Range	Sport	Population	Study duration	Total number of timepoints participants could be monitored for (Range)
Aerenhouts et al. (2013) [28]	60 Male *n* = 31 Female *n* = 29	Male and Female	12 to 18 years	Sprint (60–400m flat and hurdle) athletes	Top 10 in Flemish athletics league	1.5 years	4 (4)
Bidaurrazaga-Letona et al. (2014) [29]	38	Male	U11 to U16	Football	Professional club	4 years	8 (NA)
Bishop et al. (2020) [30]	18	Male	U23	Football	Academy	1 season	3 (3)
Booth et al. (2020) [31]	147	Male	U15 to U18	Rugby League	Elite club	2 seasons	6 (NA)
Carvalho et al. (2014) [32]	33	Male	10 to 15 years	Football	Professional club	4 years	8 (NA)
Casserly et al. (2020) [33]	15	Male	U18 to U20	Rugby Union	Academy	3 seasons	3 (3)
Deprez et al. (2014) [34]	162	Male	10 to 14 years	Football	Academy	5 years	14 (3–14)
Deprez et al. (2015) [35]	555	Male	7 to 17 years	Football	Academy	7 years	15 (3–15)
Deprez et al. (2015) [36]	42 (2 year sub sample *n* = 21, 4 year sub sample *n* = 21)	Male	7 to 17 years	Football	Academy	4 years	3 (3)
Dobbin et al. (2019) [37]	197	Male	17.3 ± 1.0 years	Rugby League	Academy	2 seasons	8 (NA)
Elferink-Gemser et al. (2006) [38]	217 Male *n* = 110 Female *n* = 107	Male and Female	12 to 19 years	Field Hockey	Talent development programme	3 seasons	3 (1–3)
Elferink-Gemser et al. (2007) [39]	126	Male and Female	12 to 16 years	Field Hockey	Talent development programme	4 seasons	3 (1–3)
Forsman et al. (2016) [40]	288	Male	12 to 14 years	Football	Club	1 year	3 (2–3)
Francioni et al (2018) [41]	33	Male	U14	Football	Club	1 season	6 (NA)
Fransen et al. (2017) [42]	2228	Male	5 to 19 years	Football	Academy	6 years	14 (1–14)
Ingjer (1992) [43]	7	Male	13 to 17 years	Skiers	National	9 years	3–6 times annually (NA)
Kramer et al. (2016) [44]	190, 123 used for the multilevel modelling Male *n* = 113 Female *n* = 83	Male and Female	U14 to U16	Tennis	Talent development programme	7 years	9 (2–3 per year)
Kramer et al. (2016) [45]	256	Male	10 to 15 years	Tennis	Talent development programme	5 years	10 (median 3)
Leyhr et al. (2018) [46]	1134	Male	U12 to U18	Football	TID programme	3 years	4 (4)
Leyhr et al. (2020) [47]	737	Female	U12 to U18	Football	TID programme	10 years	4 (2–4)
López-Plaza et al. (2019) [48]	13 (7 male and 6 female)	Male and Female	13.41 ± 0.47 to 15.64 ± 0.66	Paddlers	National	3 years	3 (NA)
Madsen et al. (2018) [49]	30	Male	U15 to U19	Badminton	National	2 years	3 (NA)
Matthys et al. (2013) [50]	94	Male	U14 to U16	Handball	National, academy and club	3 seasons	3 (1–3)
Philippaerts et al. (2006) [51]	76	Male	10 to 18	Football	Elite, sub-elite and non-elite	5 years	5 (4–5)
Roescher et al. (2010) [52]	130	Male	14 to 18	Football	TID programme	6 years	5 (1–4)
Saward et al. (2020) [53]	2875	Male	8–19	Football	Academy	11 years	NA (1–24)
te Wierike et al. (2014) [54]	36	Male	14 to 19	Basketball	Academy	2 seasons	6 (1–6)
Till et al. (2013) [55]	81	Male	U13 to U15	Rugby League	Performance pathway	4 years	3 (3)
Till et al. (2014) [56]	81	Male	U13 to U15	Rugby League	Performance pathway	4 years	3 (3)
Till et al. (2014) [57]	75	Male	U14 to U20	Rugby League	Academy	6 years	12 (NA)
Till et al. (2015) [58]	65	Male	U16 to U19	Rugby League	Academy	6 year	4 (4)
Till et al. (2016) [59]	81 (25 for longitudinal)	Male	U17 to U19	Rugby League	Academy	3 years	3 (1–3)
Till et al. (2017) [60]	51	Male	U13 to U15	Rugby League	Performance pathway	4 years	3 (3)
Valente-Dos-Santos et al. (2012) [61]	83	Male	11 to 18	Football	Club	5 years	5 (3–5)
Valente-Dos-Santos et al. (2012) [62]	135 (83 learning, 52 test data set)	Male	11 to 18	Football	Club	5 years	5 (3–5)
Valente-Dos-Santos et al. (2012) [63]	135 (83 learning, 52 test data set)	Male	11 to 18	Football	Club	5 years	5 (3–5)
Valente-Dos-Santos et al. (2014) [34]	135 (83 learning, 52 test data set)	Male	11 to 18	Football	Club	5 years	5 (3–5)
Waldron et al (2014) [64]	13	Male	U15 to U17	Rugby League	Academy	3 seasons	3 (3)
Wright & Atkinson (2019) [65]	14	Female	12.1 ± 0.9 years	Football	Centre of excellence	3 years	4 times annually (3–4 annually)
Zhao et al (2020) [66]	21	Male	12–14	Swimming and Racket sports	Elite sport school	2 years	5 (5)

### Data collection

#### Dependent variables

In total 12 physical and sport specific qualities were assessed across the articles, shown in Table 3. S1 Table provides further detail on the dependent variables of each study. Aerobic capacity (*n* = 26; 65%), speed (*n* = 24; 60%), muscular power (*n* = 23; 58%) and anthropometrics (*n* = 22; 55%) were the most frequently assessed physical qualities. Three (8%) studies reported the use of sport specific tests which compromised of dribbling [50, 61] and paddling [48] speed. Two (5%) articles constructed latent variables to be used as dependent variables from latent growth models [40] and principle component analysis [44].

**Table 3:** The dependent variables assessed in the articles.

Physical Quality	n
Aerobic capacity	26
Anaerobic capacity	2
Anthropometrics	22
Balance	2
Body composition	10
Change of direction	16
Flexibility	4
Muscular power	23
Repeated sprint	5
Speed	24
Sport specific performance	3
Strength	9

#### Independent variables

The independent variables are shown in Table 4, with the independent variables for each study show in S1 Table. The independent variables are summarised as developmental, physical, psychological, sport and temporal variables. Temporal variables were the most frequently used (*n* = 44) independent variables incorporated in all studies as categorical variables (i.e., time, season period and age grade as categorical variables and age and training age as continuous). Four (10%) studies included two temporal variables [32, 37, 39, 40]. The inclusion of physical, developmental and sport related variables were next most common with a total of 34, 20 and 21 respectively. Five studies reported retrospective analysis including variables relating to career progression / attainment in combination with the change over time [46, 47, 52, 59, 60]. Psychological variables were the least common included on only 2 occasions [38, 40].

**Table 4:** The independent variables used to evaluate the development of physical qualities.

Independent variable	n
Temporal	
Time (categorical)	13
Season period	3
Age (continuous)	19
Age grade (categorical)	6
Training age	3
Developmental	
Relative age	2
Maturation	11
Growth	1
Career progression / attainment	6
Sport	
Position	5
Standard	8
Training load	4
Gender	1
League ranking	1
Technical	1
Tactical	1
Physical	
Power	5
Height	9
Body mass/composition	11
Balance	2
Motor skills	1
Anthropometric	1
Aerobic capacity	4
Change of direction	1
Psychological	
Motivation	2
Competence	1

#### Number of observations per participant

The mean number of maximum observations for participants per study was six, with a median of five. The range of participant observations within the literature is 1 to 24. Ten (25%) of the articles stated that they employed a mixed-longitudinal design [32, 34, 41, 42, 44, 45, 53, 54, 61, 63] or that some participants in the analysis completed fewer than three observations [38, 39, 50, 52]. Six articles also reported a range of participant observations of three or more [34, 36, 47, 51, 65, 67]. The remaining articles either confirmed that all participants were observed at all time points [13, 28, 30, 33, 35, 46, 56, 58, 59, 64, 66], or the number of participant observations was not stated [29, 31, 32, 37, 42, 48, 49, 57].

#### Missing data

Thirteen studies (33%) used complete case analysis [28, 30, 36, 40, 49, 50, 55, 56, 58, 60, 65, 66, 68]. Eight studies (20%) stated within the analysis justification that the method could accommodate missing values [35, 38, 39, 44, 45, 47, 52, 54]. One study performed an analysis (Little’s missing completely at random test) to check the mechanism for missing data [40]. The remaining studies (*n* = 19; 48%) failed to address their approach to how missing data were dealt with.

### Statistical analysis

#### Analysis methods

The analysis methods used to monitor the longitudinal change in physical testing data are reported in Table 5. Regression analysis was the most common approach used in 24 studies (60%). Multilevel models, also described as hierarchical models and mixed effect models, were utilised in 20 studies (50%) using random intercepts and slopes to identify with and between participant variation. Most studies assessed the relationship between chronological age or maturation with physical qualities to identify the longitudinal development, while two studies chose to use timepoints within season or across multiple seasons [33, 37]. Polynomial regression was used to quantify development curves for maximal oxygen consumption and chronological age [43] and change in physical performance and maturation [51]. Segmented linear analysis was employed to identify an abrupt changepoint in the slope between the physical qualities and age to signify difference in the rate of development [42]. Generalised linear model was used in conjunction with a repeated measures analysis of variance (ANOVA) to accommodate for the addition of age as a covariate [66].

**Table 5:** The analysis methods used to evaluate the development of physical qualities in the identified articles.

Analytical method	n	Reference
Regression modelling		
Multilevel models	20	[28, 29, 31–35, 37, 38, 44–47, 52–54, 61–63]
Segmented linear model	1	[42]
Generalised linear model	1	[66]
Polynomial regression	2	[43, 51]
Structured equation modelling		
Latent growth modelling	1	[40]
Analysis of variance		
ANOVA	1	[57]
MANOVA	1	[36]
Repeated measures ANOVA	5	[48, 49, 58, 64, 66]
Repeated measures ANCOVA	2	[39, 50]
Repeated measures MANOVA	4	[55, 56, 59, 60]
Repeated measures MANCOVA	1	[55]
Non-parametric		
Friedmans analysis of variance	3	[30, 41, 48]
Х^2^ tests	1	[43]
Magnitude based inferences (within and between)	1	[65]

ANOVA, analysis of variance; MANOVA, multiple analysis of variance

ANOVA and multivariate analysis of variance (MANOVA) techniques were also commonly implemented to identify the longitudinal change in physical qualities. Two studies used an independent groups ANOVA [57] and MANOVA [36] to identify differences in physical qualities between seasons. Nine studies employed repeated measures ANOVA (*n* = 5; 13%) and MANOVA (*n* = 4; 10%). Repeated measure analysis of covariance (ANCOVA) and multiple analysis of covariance (MANCOVA) were used in 3 articles (8%) to account for covariates (e.g., maturation, playing standard or age) within the analysis, typically identifying differences between timepoints or age grades. The Friedman test and magnitude-based inferences were also used to identify the differences in physical qualities between timepoints. X^2^ was also applied to identify between participant differences in the development of maximal oxygen consumption following the use of a second order polynomial regression [43].

#### Justification for statistical approaches

Nineteen articles (48%) provided justification for the statistical analysis undertaken within the studies. The most common reasons for model selection included the accommodation for variable spacing between observations and different observation numbers (*n* = 7; 18%) [33, 34, 38, 44, 45, 52, 54], based on previous research (*n* = 4; 10%) [42, 46, 51, 65] and accommodation for inter-individual variation (*n* = 5; 13%) [34, 47, 61, 63, 67]. Other reasons included within and between season differences, comparison of sub-groups over time [39], non-normal data [30] and longitudinal design [28]. Methods for supporting the justification included referencing a journal article (*n* = 14; 35%) [29, 31–34, 42, 46, 47, 51, 52, 61–63, 65], the MLwiN software (*n* = 5; 13%) [36, 38, 44, 45, 63], books (*n* = 2) [28, 54] and Hopkins website (*n* = 1) [65].

#### Addressing statistical assumptions

Fifteen articles stated they had assessed the statistical assumptions required. For multivariable regression analysis, multicollinearity was assessed through tolerance checks (*n* = 5; 13%) [34, 36, 61–63] and variance inflation factor (*n* = 5; 13%) [34, 36, 61–63]. Residuals of the models were also assessed through visual inspection of Q-Q plots (*n* = 1; 3%) [37] and the distribution of the residuals against predicted values (*n* = 1; 3%) [29]. Normality was assessed in eight studies, Kolomorogov-Smirnov (*n* = 3; 8%) [41, 50, 57], Shapiro-Wilks (*n* = 2; 5%) [30, 48], Mahalanobis distance (*n* = 1; 3%) [40] and no specific test stated [64]. Sphericity of data was assessed in one study [64].

#### Assessing model fit

Thirteen studies could not be assessed for model fit. Of those that could, 13 outlined how model fit was assessed by Akiake Information Criteria (*n* = 4; 10%) [29, 42, 52], a test data set (*n* = 3; 8%) [61–63], log likelihood ratio (*n* = 5; 13%) [28, 44–47], restricted maximum likelihood (*n* = 3; 8%) [28, 29, 32], Chi squared, standardised root mean square residual, root mean square error of approximation, comparative fit index and Tucker-Lewis index (*n* = 1; 3%) [40].

#### Alignment with fitness testing theoretical and temporal challenges

The ability for the statistical methods used within the current research to assess the development of physical qualities to align with theoretical and temporal challenges of data collection and analysis is summarised in Table 6. The appropriateness of the statistical methods employed in individual studies can be found in Table 7. Through a qualitative assessment, each analysis method was evaluated against the challenges faced through longitudinal fitness testing data. The overall success for each analysis method in fulfilling these challenges is presented as the percentage of studies which met them. The methods are presented in order of overall success of the analytical methods summarised through an average percentage across all challenges. It should be noted that this is not an extensive list of all statistical methods, but rather a summary and qualitative evaluation of those currently employed in the longitudinal assessment of physical testing data. Consequently, it is possible that some statistical methods could have been employed to align with physical testing requirements but were not. For example, latent growth modelling can be specified to be non-linear by assigning different weightings for the loading variables between timepoints [18], however, Forsman et al. [40] employed it with equal weighting for the loading variables, therefore observing a linear change over time.

**Table 6:** A qualitative assessment of the ability of statistical methods to meet the theoretical and temporal challenges faced when evaluating longitudinal fitness testing data.

		Theoretical challenges			Temporal challenges				Summary
Analysis method	n	Multi-dimensional	Non-linear Change	Group and individual athlete change	Time variant and time invariant	Missing data and unbalanced designs	Time is included as a continuous variable	Repeated measures	Average agreement with theoretical and temporal challenges
Multilevel linear models	20	90%	75%	65%	75%	100%	90%	100%	85%
Latent growth modelling	1	100%	0%	0%	100%	0%	100%	100%	57%
Repeated measures MANCOVA	1	100%	0%	0%	100%	0%	0%	100%	43%
Polynomial regression	2	0%	100%	0%	0%	100%	100%	50%	50%
Repeated measures ANCOVA	2	100%	0%	0%	100%	0%	0%	100%	43%
Generalised linear model	1	100%	0%	0%	100%	100%	0%	0%	43%
Segmented linear model	1	0%	100%	0%	0%	100%	100%	0%	43%
Repeated measures MANOVA	4	75%	0%	0%	75%	0%	0%	100%	36%
Repeated measures ANOVA	4	0%	0%	0%	0%	0%	0%	100%	14%
Friedmans analysis of variance	3	0%	0%	0%	0%	0%	0%	100%	14%
Magnitude based inferences (within and between)	1	0%	0%	0%	0%	0%	0%	100%	14%
ANOVA	1	0%	0%	0%	0%	0%	0%	0%	0%
MANOVA	1	0%	0%	0%	0%	0%	0%	0%	0%
Х2 tests	1	0%	0%	0%	0%	0%	0%	0%	0%

ANOVA, analysis of variance; MANOVA, multiple analysis of variance. The data presented show the number of studies that align with the theoretical and temporal challenges for each statistical analysis method as a percentage of the total studies that apply the method.

**Table 7:** Study information and individual qualitative analysis.

Author	Physical qualities assessed	Dependent variable	Statistical analysis method	Multi-dimensional	Non-linear Change	Group and individual athlete change	Time variant and time invariant	Missing data and unbalanced designs	Time is included as a continuous variable	Repeated measures
Aerenhouts et al. (2013) [28]	Anthropometrics, body composition	Time	Multilevel modelling							
Bidaurrazaga-Letona et al. (2014) [29]	Anthropometrics, muscular power, speed, change of direction	Age, maturation	Multilevel modelling							
Bishop et al. (2020) [30]	Muscular power	Time	Friedmans analysis of variance							
Booth et al. (2020) [31]	Aerobic capacity, muscular power, muscular strength, change of direction	Rugby league training age, resistance training age	Multilevel modelling							
Carvalho et al. (2014) [32]	Anthropometrics, aerobic capacity	Age, maturation, season period	Multilevel modelling							
Casserly et al. (2020) [33]	Muscular power, speed, aerobic capacity	Time, position, baseline and change in body mass	Multilevel modelling							
Deprez et al. (2014) [34]	Aerobic capacity	Age, height, body composition, balance, maturation	Multilevel modelling							
Deprez et al. (2015) [35]	Muscular power	Age, anthropometrics, body composition, balancing, moving sideways, jumping sideways	Multilevel modelling							
Deprez et al. (2015) [36]	Anthropometrics, aerobic capacity	Standard, time	MANOVA							
Dobbin et al. (2019) [37]	Anthropometrics, change of direction, speed, muscular power, aerobic capacity	Season phase, playing year, playing position, league ranking, anthropometrics, physical characteristics	Multilevel modelling							
Elferink-Gemser et al. (2006) [38]	Aerobic capacity	Age, gender, standard, body composition, training load, motivation	Multilevel modelling							
Elferink-Gemser et al. (2007) [39]	Anthropometrics, body composition, speed, repeated sprint, change of direction, aerobic capacity	Time, standard, age	RM ANCOVA							
Forsman et al. (2016) [40]	Speed, change of direction	Time, level, growth, age, motivation, competence	Latent growth models							
Francioni et al (2018) [41]	Anthropometrics, muscular power, speed	Time	Friedmans analysis of variance							
Fransen et al. (2017) [42]	Anthropometrics, muscular strength, flexibility, change of direction, speed, power, aerobic capacity	Age	Segmented linear models							
Ingjer (1992) [43]	Aerobic capacity	Age	Polynomial regression and chi squared							
Kramer et al. (2016) [44]	Anthropometrics, speed, muscular power, change of direction	Age, maturation, standard	Multilevel models							
Kramer et al. (2016) [45]	Speed	Age, standard, body mass, countermovement jump	Multilevel models							
Leyhr et al. (2018) [46]	Speed, change of direction	Period in years after first assessment, adult performance level, relative age	Multilevel models							
Leyhr et al. (2020) [47]	Speed, change of direction	Period in years after first assessment, adult performance level	Multilevel models							
López-Plaza et al. (2019) [48]	Anthropometrics, body composition, sport specific performance	Time	Repeated measures ANOVA and Friedmans analysis of variance							
Madsen et al. (2018) [49]	anthropometrics, speed, power, sport specific performance	Time	Repeater measures ANOVA							
Matthys et al. (2013) [50]	Anthropometrics, body composition, flexibility, aerobic capacity, muscular power, muscular strength, aerobic performance, sport specific performance, speed	Time, standard, maturity offset	Repeated measures ANCOVA							
Philippaerts et al. (2006) [51]	Anthropometrics, balance, muscular strength, muscular power, flexibility, speed, aerobic capacity, anaerobic capacity	Maturation	Polynomial regression							
Roescher et al. (2010) [52]	Aerobic capacity	Age, height, lean body mass, level, percentage of body fat, training load, playing position	Multilevel models							
Saward et al. (2020)	Anthropometrics, muscular power, speed, change of direction, aerobic capacity	Position, age, career progression	Multilevel models							
te Wierike et al. (2014) [54]	Repeated sprint	Age, height, body composition, vertical jump and interval shuttle test	Multilevel models							
Till et al. (2013) [55]	Anthropometrics, body composition, muscular power, speed, change of direction, aerobic capacity	Age, chronological age, maturation	Repeated measures MANOVA and MANCOVA							
Till et al. (2014) [56]	Anthropometrics, body composition, muscular power, muscular strength, speed, change of direction, aerobic capacity	Season period, age	T-test and ANOVA							
Till et al. (2014) [57]	Anthropometrics, body composition, muscular power, speed, change of direction, aerobic capacity	Age, relative age, maturation	Repeated measures MANOVA							
Till et al. (2015) [58]	Anthropometrics, body composition, muscular power, muscular strength, speed, aerobic capacity	Age	Repeated measures ANOVA							
Till et al. (2016) [59]	Anthropometrics, body composition, muscular power, muscular strength speed, aerobic capacity	Age, career progression	Repeated measures MANOVA							
Till et al. (2017) [60]	Anthropometrics, body composition, muscular power, speed, change of direction, aerobic capacity	Age, career progression	Multilevel models							
Valente-Dos-Santos et al. (2012) [61]	Repeated sprint, change of direction, muscular power, aerobic capacity	Age, maturation, position, body composition, stature, training load, sport specific	Multilevel models							
Valente-Dos-Santos et al. (2012) [62]	Aerobic capacity	Age, maturation, body composition, training age, stature	Multilevel models							
Valente-Dos-Santos et al. (2012) [63]	Repeated sprint	Age, maturation, aerobic capacity, power, body composition, training experience, stature	Multilevel models							
Valente-Dos-Santos et al. (2014) [34]	Change of direction	Age, maturation, body composition, stature, aerobic capacity, power, training load	Multilevel models							
Waldron et al (2014) [64]	Anthropometrics, muscular power, speed, aerobic capacity	Age	Repeated measures ANOVA							
Wright & Atkinson (2019) [65]	Speed, muscular power, repeated sprint	Time	Within and between participant magnitude-based inferences							
Zhao et al (2020) [66]	Anthropometrics, aerobic capacity, muscular strength	Time, sport	Generalised linear model and repeated measures ANOVA							

*ANOVA*, analysis of variance; *MANOVA*, multiple analysis of variance. Green shading indicates the analysis method accommodated for the theoretical and temporal challenges faced, while those in red failed to do so.

## Discussion

This is the first systematic methodological review to identify and qualitatively evaluate the application of statistical methods used to analyse longitudinal physical qualities data within youth athletes. The present study identified 40 articles which met the inclusion criteria. In total, 13 sports were assessed across a range of levels from elite schools, clubs, academies and talent development systems. The mean number of participants across the included studies was 264 (range; 7 to 2875) completed over a mean period of 4 years/seasons (range; 1 to 11 years). Studies more frequently used male participants (n = 32), with mixed (n = 5) and female (n = 2) cohorts more infrequent. The mean number of maximum possible timepoints within each study was 6 (range; 1 to 24). In total 12 physical and sport specific qualities were identified as dependent variables while independent variables were be grouped into developmental, physical, psychological, sport and temporal categories. Several statistical methods were used including regression, structural equation modelling, ANOVA, non-parametric and magnitude-based inferences approaches. When aligning the statistical methods employed within these studies with the theoretical underpinnings and temporal challenges of the longitudinal assessment of physical qualities, the qualitative assessment identified a varying degree of success in their alignment.

### Multidimensional analysis

The development of physical qualities is complex requiring multiple factors of an individual (e.g., sex, chronological and biological development [44, 69]). This can be further compounded by sport specific factors (e.g. position or playing level [33, 59]). The complexity of the development of physical qualities is supported by the variety of independent variables identified within the articles (Table 4). It is therefore imperative that multiple factors that account for and explain the within and between participant differences in the development of physical qualities are accommodated within such analysis.

Whilst some studies considered a multivariable (i.e. multiple independent/predictor variables) approach to dealing with the complexity of the development of physical qualities, several were limited by the inability to incorporate this into the statistical analysis with the method selected (i.e. ANOVA, X^2^, Friedmans and magnitude-based inferences). Consequently, the development of physical qualities was only assessed as a result of time (age grade or timepoint). However it is also apparent that a single independent variable was used where a multivariable analysis could be applied, with multilevel [28, 31] and segmented linear modelling [42]. This is a consequence of the specific research questions, rather than limitations of the analysis methods used. For example, Booth et al. [31] used multilevel models to identify the influence of different training ages by including each type of training age individually within their own model. As such Booth et al. [31] were able to identify the magnitude of change over time (regression slope) for each individual training age type but may have missed potential inter-relation effects between them.

The use of methods which allow for the incorporation of multiple independent variables include repeated measure ANCOVA and MANCOVA, multilevel models and latent growth modelling. The addition of covariates to time allows researchers and practitioners to understand how the characteristics of individuals effect the dependent variable to provide greater understanding of within and between participant changes in physical qualities. Covariates can be implemented to identify between group differences as categorical variables (e.g. position [33, 53, 63]) or as continuous predictor variables (e.g. maturation [44, 61, 62, 67]).

The inclusion of covariates can allow for the exploration of causal pathways through the identification of moderators and mediators. Moderation, similar to interaction, is when the effect of the independent variable (i.e. age or time) vary between groups of athletes or athletes with different characteristics. This differs from an interaction as moderation focuses on the individual effect of the independent variable and the covariates rather than the joint effect as an interaction does. While there are several articles that incorporate interactions in the model with categorical (e.g. career attainment [46, 47, 52, 59, 60]) or continuous (e.g. maturation [29, 32]) covariates, only Forsman et al. [40] currently assess moderation effects through latent growth models. The influence of covariates on the main independent variable (time) is assessed by regressing them on the slope and reporting the sperate slope and covariate effects. Similarly, no articles appropriately assess mediation. Mediators help to understand causal pathways and why an exposure leads to a particular outcome. While Casserly et al. [33] suggest they have performed a mediation analysis on the effect of change in body mass on physical qualities over time, only one model calculating the total effect is used rather than the required two models to differentiate the direct and indirect effects of the independent variable and mediators [70, 71]. Therefore, the use of the term mediator is incorrect and the change in body mass should be considered a predictor variable. Although causal pathways may seem beneficial in understanding the complexity of the development of physical qualities, there is currently limited assessment of both moderation and mediation effects within the current research. Such analysis could provide further insight into relationships between variables such as maturation, body mass and muscular strength which were highlighted in a recent article that did not meet the search criteria [72] and should therefore be considered in the future.

Furthermore, there is a paucity of research addressing multivariate outcomes. This may be of particular importance due to the multicollinearity of physical testing measures [73]. The inclusion of a correlation structure between dependent variables can account for collinearity by effectively assessing patterns between them, rather than including outcome measures as predictor variables. MANOVA [55, 56, 59, 60] and MANCOVA [55] are the most common application of multivariate analysis, however they are both limited to the comparison of mean differences between groups. Latent growth modelling, on the other hand, provides a more adaptable process which can compare the relationship between not only mean differences (i.e. intercepts), but also the change (i.e. slope) in a bivariate manor [40]. Latent growth models can therefore examine if change processes in dependent variables are related over time [74].

Future research should consider collecting multiple variables within the research design which may explain the rate of change in physical qualities and look to utilise statistical approaches (i.e., ANCOVA, MANCOVA, multilevel modelling and latent growth modelling) that can account for such a design. Consideration of the variable type, multiple dependent or independent, will dictate the analysis method used. Latent growth models should be preferred for multivariate analysis while ANCOVA, MANCOVA and multilevel models can also be considered for multivariable analysis. Mediators for the development of physical qualities should also be considered within future research through methods which are not currently used within the literature such as latent change score models.

### Identification of non-linear change

Due to the influence of individual (i.e. growth, maturation, training age) and performance (i.e. periodisation) factors, the rate at which physical qualities develop over time is proposed to be non-linear [75]. For example, growth and maturation have been identified to influence both timing and tempo of the peak changes in physical qualities [75, 76] and the rate at which qualities improve across a season are not consistent [31]. If only a linear development in physical qualities is considered this may therefore result in an over- or under-estimation at a given age or timepoint. Thus, the potential for non-linear improvements in physical qualities should be considered during analysis to provide a more accurate estimation of the development of physical qualities.

Between group comparisons (i.e., ANOVA variations, Friedmans and magnitude-based inferences), X^2^ and latent growth modelling failed to assess non-linearity of the development of physical qualities. Although between group comparisons, presented in the form of a mean for each timepoint, may show a non-linear relationship this is not statistically tested and the continuity of the development process is reduced to a straight line [77]. For example, ANOVAs identify variation from the grand mean and the subsequent post-hocs compare the differences between two groups as a linear difference. Similarly the *X*^2^ statistic identifies a difference between distributions and is used in tandem with a polynomial regression which is first identifies a non-linear distribution [43]. It should be noted that although latent growth models were only used in a linear fashion by Forsman et al. [40], the slope loading factors can be specified to be curvilinear such as a quadratic or can be unspecified [18]. Unspecified factor loadings rely on the observed data to provide an estimation of the rate of change, providing a more exploratory assessment rather than confirmatory.

Only regression methods identified the non-linear development of physical qualities. Polynomial regression, multilevel models and segmented modelling were used. Smoothing polynomials and mathematical fitting used within polynomial and multilevel models can identify the non-linear development of physical qualities by testing the model fit (e.g. significant terms, improved Akike Information Criteria or *X*^2^ statistic). For example, Carvalho et al. [32] found both quadratic and cubic terms for age significant (p ≤ 0.01) in height and Yo-Yo intermittent recovery test level 1 models suggesting a non-linear relationship between time and physical qualities was present. Non-linear terms were not included within all articles making use of multilevel models [31, 33, 37, 54], while others included the assessment of non-linear terms in the methods although they failed to reach significance (e.g. Valente Dos Santos et al. [61, 63] and skeletal age). In a similar fashion to the unspecified latent growth model, non-smoothed regressions can also be applied without the preselection of a growth model allowing for a more exploratory approach to identifying the rate of change [77]. Unlike smoothed approaches, segmented regression analysis identifies a “break point” at which the rate of development changes [42]. Due to the detection of a “break point”, this analysis is of particular benefit for the identification of potentially important transition periods in development.

It is therefore suggested that researchers should consider the type of relationship their data may observe prior to selecting the analysis method. If the data is thought to follow a previous hypothesis, the non-linearity of the relationship can be specified and confirmed with multilevel models and latent growth models. If an exploratory approach is required to identify the rate of development more flexible non-smoothed polynomial regression and unspecified latent growth models would be preferred. The choice between these two methods may be dictated by the number of observation points collected with latent growth models only requiring three time points while a high measurement frequency is required for polynomial models [77]. Finally, research questions aiming to identify a specific point in the rate of change can adopt segmented regression.

### Group and individual athlete change

The separation of group estimations of change and individual athlete change is important for understanding the variability in the rate of physical development. If change is not considered on the individual level then the development of physical qualities is assumed to be the same for all athletes based on the group change. However, this is known not to be the case with the timing and tempo of development in youth athlete varying depending upon multiple factors such as maturation [78, 79]. In order to understand the range in the rate of development of physical qualities in youth athletes’ statistical methods should therefore account for the distinction between the group and individual change.

Only multilevel models and latent growth models were identified to consider different levels of athlete change within the current literature. Both independent and repeated measure group tests such as ANOVA, ANCOVA, MANOVA, MANCOVA and magnitude-based inferences only assess variation among and between groups failing to consider the potential for differences in the rate of development between athletes. Multilevel models on the other hand provide the opportunity to demonstrate the variance within clusters of the data as a result of repeated observations [80]. This can be divided into random intercepts (i.e. the between participant variation in outcome), and slopes (i.e. the difference between the individual best fit gradient and the group best fit gradient). Random slopes can therefore provide an understanding of the variation in the rate of development of physical qualities by comparing the slope of each individual to the group estimate. Twelve studies incorporated random slopes allowed to vary through constant time variables of chronological age [34, 35, 44, 45, 53, 54, 61–63], skeletal age [61–63, 67] and time [46, 47], while Roescher et al. [52] considered the inclusion of random slopes but failed to improve the model fit and were therefore removed. Latent growth models are similar providing variances for both the intercept and slope for the individual change [18].

Although separating the individual and group difference in change is important, the application and reporting of this challenge was limited. While Forsman et al. [40] state the latent growth model provides variances for the slope, they were not reported. Furthermore, Dobbin et al. [37] suggested random slopes were used but failed to state for which variable(s) and report the variance as a result. To optimise the interpretation and application of the variability of the slopes by practitioners both latent growth models [18] and hierarchical models [80] should be used to determine the between athlete variation in change in addition to the average group change, making sure to report the methods and statistics correctly.

### Time varying and invariant variables

When a multidimensional approach to the assessment of longitudinal physical testing is taken, factors can remain constant over the period of testing, (i.e. time invariant, e.g. gender, maturity status, relative age quartile), while others will vary (i.e. time varying, e.g. body mass, physical performance in other tests). Time invariant covariates are used to identify differences between groups of athletes, meanwhile time varying covariates determine between athlete differences and also within athlete change. It is therefore important that the analysis method can incorporate both time varying and invariant variables to establish the longitudinal development of physical qualities.

Methods which fail to take a multidimensional approach to the longitudinal analysis of physical testing data (i.e. segmented linear models, polynomial regression, repeated and independent ANOVA, MANOVA, Friedmans, magnitude-based inferences and X^2^) did not include time invariant factors within the analysis. Such approaches should therefore not be considered when trying to effectively outline the multidimensional development of physical qualities in youth athletes.

Multiple analysis methods including repeated measures ANCOVA, MANOVA, MANCOVA and generalised linear models were shown to be able to incorporate time invariant covariates (e.g. maturation groups [55, 56]; playing level [39]) along with time. Such methods can also make use of time varying covariates although only one study chose to do so with a repeated measures ANCOVA [39] potentially highlighting their application to observe between group differences in change rather than individual athlete development. In contrast to this, latent growth models and multilevel models both included time varying and invariant covariates. However, interpretation of the within and between effects of time varying can be difficult. Taking a grand mean centring approach with the covariate gathers both the within and between person effects and variances and represents an uninterpretable blend of the data [20, 81, 82]. It is therefore suggested that changes to the model are made to tease out the differences between the within and between differences by performing individual centring of the covariate for example [81]. While multilevel models and latent growth models benefit from the ability to incorporate time varying covariates and have done so in the current literature, caution should be taken with the interpretation of the results where modelling adjustments have been made to differentiate the within and between athlete differences. Future research should therefore consider utilising appropriate methods to enhance the understanding of the within and between person effects of covariates on the development of physical qualities.

### Missing data and unbalanced designs

Missing data is a common issue associated with longitudinal physical testing in youth athletes with reasons for missing data suggested to be injury, illness, exams and drop out [40]. Missing data can be categorised into three mechanisms; missing completely at random, missing at random and missing not at random [83, 84]. Missing completely at random is due to chance (e.g. a player misses testing session through non-sport related reason) resulting on minimal bias in the data. In comparison, missing at random resulting from an observed variable (e.g. sprint testing is recorded to have been performed on grass and not an artificial pitch) or missing not at random occurring as a result of an unobserved variable (e.g. a specific club does not want to perform a particular test from the testing battery) suggest data are missing systematically and therefore are biased. Due to the variability in participant availability, club participation and facilities, especially in the case of multi-club studies and long-term studies in talent development environments, it is inevitable that missing data will be present in datasets and researchers are able to deal with such issues [85].

The treatment of missing data can be categorised into deletion and imputation methods. Statistical methods such as ANOVA, ANCOVA, MANOVA and MANCOVA require complete datasets for analysis. All studies identified within this review choosing to perform listwise deletion (i.e. excluding all participants with missing data, when missing data was present). Without confirmation of the missing data mechanism being missing at random, listwise deletion is likely to result in biased data with large standard errors, low statistical power and small sample sizes [83]. Furthermore, this excludes real data that could be used to provide more accurate estimates within the dataset and therefore deletion methods should be avoided in favour of imputation methods.

Although no articles employed specific imputation methods to generate complete datasets, several analyses identified within this review are robust to missing and unbalanced data. Studies using polynomial regression, generalised linear models, multilevel models, segmented linear modelling and latent growth models all employed mixed-longitudinal designs with participants completing a different number of observations. The ability for such methods to allow the number and temporal spacing of observations to vary between participants was highlighted within the rational for model selection within numerous articles [33, 34, 38, 44, 45, 52, 54]. These methods make use of likelihood estimation to impute the missing data. For example, multilevel models employing a full-likelihood method offering greater flexibility with missing data mechanisms [19, 86]. It should be noted that imputation methods, including maximum likelihood estimation, are only unbiased under missing completely at random and missing at random mechanisms [83]. Although this is the case, only Forsman et al. [40] performed an assessment of the missing data through Little’s MCAR test and assessment of population frequencies to confirm the data was missing completely at random or missing at random. The use of methods that employ likelihood estimation may therefore be preferred when analysing physical testing data where complete cases are not present, however future research should also consider the missing data mechanisms as best statistical practice.

Within physical testing data there is also the possibility of the occurrence of no data rather than missing data. For example, a dataset collected over several years in an elite sport academy may observe players that undergo de-selection or decide to move away from the sport and therefore do not have missing data but rather no data points collected once they leave the system. While the concept of no data is raised by Borg et al. [84], there is very little consideration for its handling within the current literature. Where studies have reported development curves across the age of the sample collected (e.g. Seward et al. [53]) and the results from multiple athletes are required to provide an estimate across the age range, the model is likely to be a reflection of the selection bias for that development system rather than the true change in physical qualities. This is a limitation of modelling by age when the possibility of no data and changes to the treatment of time could minimise the effects of no data due to reasons such as drop out and de-selection by aligning timepoints and analysis with selection cycles (i.e. analyse data across a season).

### Treatment of time

Due to the time unstructured nature of physical testing data collection, it is important to consider how time is included within the analysis. Time is typically treated in three ways in physical testing research, binned into timepoints (i.e. season number or period of the season) or age grades (e.g. under-15, -16 and -17) or continuously as some form of age (chronological, skeletal or relative to peak height velocity). Due to these differences in how time is treated, there are implications for the interpretation of the results and application of findings within practice.

Multilevel models and polynomial regression both incorporate time as a continuous variable within the analysis. Consequently, variable periods of time between testing can be incorporated in the analysis with the change in physical qualities relating to a change in age or maturation status rather than the difference between fixed timepoints. Due to the challenges faced with data collection of physical testing data these methods may therefore be preferred to accommodate variable spacing between timepoints.

Alternatively, between group comparisons (ANOVA variations, Friedmans and magnitude-based inferences), multilevel models, latent growth models and generalised linear models have grouped data based on age grade or timepoint. In the case of testing across a single season [30, 41] or club [33, 57, 59, 64] studies testing at one period or with standardised testing dates provide the development of physical qualities over a known time period. However, multi-season or multi-club studies can provide a wider testing window for each time points which could be defined as several months [37, 40]. The time between testing points may therefore need to be considered within the analysis to identify any observed effect. Furthermore the categorisation of continuous data such as age, can hide relationships between dependent and independent variables and limits the generalisability of data [72, 87]. A continuous time variable should be considered as a covariate or interaction term when time is binned within the analysis (e.g. the inclusion of age as a covariate [39, 40]) to avoid lost relationships within the data. While this provides a solution for one of the challenges faced when categorising data for analysis, future research should be aware of the limitations and consider its appropriateness for the analysis being performed.

### Repeated measures

Longitudinal research involving repeated observations results in a non-independence of data which must be accounted for. Independent comparisons (i.e., ANOVA, MANOVA, magnitude-based inferences and X^2^) assume independence of observations do not accommodate repeated measures presuming that participants only provide one observation to the analysis. Segmented linear models and polynomial regression also fail to account for repeated measures, with no way of controlling for clustering with the analyses used (e.g. segmented R package does not incorporate random effects in the model structure [42, 88]). As such it is suggested that they are not appropriate for use within the longitudinal assessment of physical testing data.

The most popular analysis method, multilevel models, account for clustering through the incorporation of player as a random effect with their ability to accommodate repeated measures [28]. Repeated measure ANOVA and MANOVA incorporate an addition level of variability (subject variability) in comparison to the independent tests. This consideration for the repeated observations results in a smaller error and therefore more powerful statistical test. Latent growth models takes into account both he means and covariances of repeatedly measured variables [18] while repeated measure difference tests account for the dependency through the consideration of the within subject variability into the test statistic. Therefore, the use of multilevel models, repeated measure ANOVA and MANOVA and latent growth models should be used in longitudinal research to account repeated observations within the data.

### Methodological considerations

Generally, the assessment of model assumptions and checking of fit was not well reported throughout the current literature. On the other hand, several authors provide detailed accounts of the checks performed during analysis which should be used to guide future reporting [29, 40]. Due to its link to the challenge of incorporating a multidimensional approach to the development of physical qualities, future research should take extra caution surrounding the risk of collinearity between variables and the consequences within analysis, such as inflated standard errors. For example, Dobbin et al. [37] included all physical testing data in each model, however data within the same cohort would suggest that these may breach multi-collinearity [73]. The use of latent growth models could be used in this instance to create latent variables from tests which capture similar information [40] or authors should conduct a test of multi-collinearity (e.g. variance inflation factor [34, 36, 61–63]), removing variables which breach this assumption and share similar variance.

### Limitations

While the current review provides a thorough assessment of the analysis methods employed within longitudinal physical testing research it is not an extensive evaluation of the possible analyses methods that could be used and their implementation. Consequently, it is possible that an alternative analysis not currently employed within the current body of literature may address the theoretical and temporal challenges in a superior way to those presented. Secondly, unlike the multifactorial aetiology [89] and complex systems [5] theoretical underpinnings of injury, there is currently no comparative frameworks for the development of physical qualities. Therefore, potential key challenges were identified through the authors understanding subject area. The theoretical underpinnings may not be conclusive but provide some initial considerations for researchers to base the selection of analytical methods from. Finally, the main aim of this review was to evaluate the analysis methods used for longitudinal physical testing data. It was therefore beyond the scope of this review to evaluate the empirical evidence gathered by the articles identified within this review. Further research is required to summarise the effects of variables on the development of physical qualities in youth athletes.

### Future directions

Future research assessing longitudinal physical testing data in youth athletes should build upon the current literature by utilising the theoretical and temporal challenges outlined to select appropriate statistical approaches, thus enhancing the use of the data collected. It is important that researchers first identify appropriate research questions with appropriate research designs that align to these challenges to in turn drive the selection of an appropriate analysis method.

While it is clear that some methods are inappropriate for longitudinal physical testing data, such as independent ANOVA and MANOVA, due to the breach of assumptions and potential risk of bias, other methods have demonstrated varying levels of competence in meeting the challenges faced from the theoretical underpinnings and temporal challenges. Multilevel models and latent growth models are identified to be the most successful. Even though multilevel models were identified to meet all the challenges in at least one article, they are not without their limitations, such as their failure to perform a multivariate analysis. Therefore, researchers should consider such limitations and potential areas of improvement in other methods when selecting statistical analysis techniques in the future.

Although it is common to see articles quantify the development of physical qualities from longitudinal data, there are very few papers which have attempted to identify and rationalise why such methods are superior. For example, Park and Schultz [18] suggest that latent growth modelling should be used to analyse longitudinal physical testing data due to several factors including its ability to address the theoretical underpinnings of between and with athlete change with multiple predictors, one of the main failings identified for multilevel modelling. Articles of this type are more common in other areas such as psychology [90, 91] and provide detail on the application of methods may be appropriate to answer specific the research questions.

## Conclusions

This qualitative systematic methodological review investigated the statistical analysis methods employed within longitudinal physical testing research to identify which methods currently make the best use of the data. To utilise the data effectively the methods selected should consider the underpinning theory of the subject area and temporal demands that occur as a result of data collection. Based on the qualitative review, statistical analysis methods using independent groups ANOVA, MANOVA and X^2^ fail to address any theoretical or temporal challenges posed by longitudinal physical testing data. On the other hand, both multilevel models and latent growth models demonstrate the ability to deal with many of the challenges presented within longitudinal physical testing. However, multilevel models and latent growth modelling still have limitations and future work is required to enhance their application. It is essential that researchers consider the challenges posed by longitudinal physical testing data when making considerations regarding the research design and selecting appropriate analysis methods to develop knowledge and understanding of the long-term physical development in youth athletes.

## Supporting information

S1 File PRISMA checklist. (DOCX)

S1 Table Study information and individual qualitative analysis. (DOCX)
