# Supplementary material for: Statistical analysis considerations within longitudinal studies of physical qualities in youth athletes: A qualitative systematic methodological review
Source: PLoS One. 2022 Jul 7;17(7):e0270336. doi: 10.1371/journal.pone.0270336 (PMC9262234; doi:10.1371/journal.pone.0270336)
Supplement: S1 Table — (DOCX) [file pone.0270336.s002.docx]

| **S1 Table. Study information and individual qualitative analysis** | | | | | | | | | | |
| --- | --- | --- | --- | --- | --- | --- | --- | --- | --- | --- |
| **Author** | **Physical qualities assessed** | **Dependent variable** | **Statistical analysis method** | **Multi-dimensional** | **Non-linear Change** | **Between and within athlete change** | **Time variant and time invariant** | **Missing data and unbalanced designs** | **Time is included as a continuous variable** | **Repeated measures** |
| Aerenhouts et al. (2013) [52] | Anthropometrics, body composition | Time | Multilevel modelling |  |  |  |  |  |  |  |
| Bidaurrazaga-Letona et al. (2014) [64] | Anthropometrics, muscular power, speed, change of direction | Age, maturation | Multilevel modelling |  |  |  |  |  |  |  |
| Bishop et al. (2020) [53] | Muscular power | Time | Friedmans analysis of variance |  |  |  |  |  |  |  |
| Booth et al. (2020) [63] | Aerobic capacity, muscular power, muscular strength, change of direction | Rugby league training age, resistance training age | Multilevel modelling |  |  |  |  |  |  |  |
| Carvalho et al. (2014) [39] | Anthropometrics, aerobic capacity | Age, maturation, season period | Multilevel modelling |  |  |  |  |  |  |  |
| Casserly et al. (2020) [54] | Muscular power, speed, aerobic capacity | Time, position, baseline and change in body mass | Multilevel modelling |  |  |  |  |  |  |  |
| Deprez et al. (2014) [41] | Aerobic capacity | Age, height, body composition, balance, maturation | Multilevel modelling |  |  |  |  |  |  |  |
| Deprez et al. (2015) [55] | Muscular power | Age, anthropometrics, body composition, balancing, moving sideways, jumping sideways | Multilevel modelling |  |  |  |  |  |  |  |
| Deprez et al. (2015) [47] | Anthropometrics, aerobic capacity | Standard, time | MANOVA |  |  |  |  |  |  |  |
| Dobbin et al. (2019) [62] | Anthropometrics, change of direction, speed, muscular power, aerobic capacity | Season phase, playing year, playing position, league ranking, anthropometrics, physical characteristics | Multilevel modelling |  |  |  |  |  |  |  |
| Elferink-Gemser et al. (2006) [45] | Aerobic capacity | Age, gender, standard, body composition, training load, motivation | Multilevel modelling |  |  |  |  |  |  |  |
| Elferink-Gemser et al. (2007) [44] | Anthropometrics, body composition, speed, repeated sprint, change of direction, aerobic capacity | Time, standard, age | RM ANCOVA |  |  |  |  |  |  |  |
| Forsman et al. (2016) [29] | Speed, change of direction | Time, level, growth, age, motivation, competence | Latent growth models |  |  |  |  |  |  |  |
| Francioni et al (2018) [38] | Anthropometrics, muscular power, speed | Time | Friedmans analysis of variance |  |  |  |  |  |  |  |
| Fransen et al. (2017) [43] | Anthropometrics, muscular strength, flexibility, change of direction, speed, power, aerobic capacity | Age | Segmented linear models |  |  |  |  |  |  |  |
| Ingjer (1992) [65] | Aerobic capacity | Age | Polynomial regression and chi squared |  |  |  |  |  |  |  |
| Kramer et al. (2016) [30] | Anthropometrics, speed, muscular power, change of direction | Age, maturation, standard | Multilevel models |  |  |  |  |  |  |  |
| Kramer et al. (2016) [36] | Speed | Age, standard, body mass, countermovement jump | Multilevel models |  |  |  |  |  |  |  |
| Leyhr et al. (2018) [35] | Speed, change of direction | Period in years after first assessment, adult performance level, relative age | Multilevel models |  |  |  |  |  |  |  |
| Leyhr et al. (2020) [34] | Speed, change of direction | Period in years after first assessment, adult performance level | Multilevel models |  |  |  |  |  |  |  |
| López-Plaza et al. (2019) [61] | Anthropometrics, body composition, sport specific performance | Time | Repeated measures ANOVA and Friedmans analysis of variance |  |  |  |  |  |  |  |
| Madsen et al. (2018) [60] | anthropometrics, speed, power, sport specific performance | Time | Repeater measures ANOVA |  |  |  |  |  |  |  |
| Matthys et al. (2013) [46] | Anthropometrics, body composition, flexibility, aerobic capacity, muscular power, muscular strength, aerobic performance, sport specific performance, speed | Time, standard, maturity offset | Repeated measures ANCOVA |  |  |  |  |  |  |  |
| Philippaerts et al. (2006) [48] | Anthropometrics, balance, muscular strength, muscular power, flexibility, speed, aerobic capacity, anaerobic capacity | Maturation | Polynomial regression |  |  |  |  |  |  |  |
| Roescher et al. (2010) [33] | Aerobic capacity | Age, height, lean body mass, level, percentage of body fat, training load, playing position | Multilevel models |  |  |  |  |  |  |  |
| Sawaerd et al. (2020) | Anthropometrics, muscular power, speed, change of direction, aerobic capacity | Position, age, career progression | Multilevel models |  |  |  |  |  |  |  |
| te Wierike et al. (2014) [37] | Repeated sprint | Age, height, body composition, vertical jump and interval shuttle test | Multilevel models |  |  |  |  |  |  |  |
| Till et al. (2013) [17] | Anthropometrics, body composition, muscular power, speed, change of direction, aerobic capacity | Age, chronological age, maturation | Repeated measures MANOVA and MANCOVA |  |  |  |  |  |  |  |
| Till et al. (2014) [58] | Anthropometrics, body composition, muscular power, muscular strength, speed, change of direction, aerobic capacity | Season period, age | T-test and ANOVA |  |  |  |  |  |  |  |
| Till et al. (2014) [59] | Anthropometrics, body composition, muscular power, speed, change of direction, aerobic capacity | Age, relative age, maturation | Repeated measures MANOVA |  |  |  |  |  |  |  |
| Till et al. (2015) [51] | Anthropometrics, body composition, muscular power, muscular strength, speed, aerobic capacity | Age | Repeated measures ANOVA |  |  |  |  |  |  |  |
| Till et al. (2016) [32] | Anthropometrics, body composition, muscular power, muscular strength speed, aerobic capacity | Age, career progression | Repeated measures MANOVA |  |  |  |  |  |  |  |
| Till et al. (2017) [31] | Anthropometrics, body composition, muscular power, speed, change of direction, aerobic capacity | Age, career progression | Multilevel models |  |  |  |  |  |  |  |
| Valente-Dos-Santos et al. (2012) [40] | Repeated sprint, change of direction, muscular power, aerobic capacity | Age, maturation, position, body composition, stature, training load, sport specific | Multilevel models |  |  |  |  |  |  |  |
| Valente-Dos-Santos et al. (2012) [66] | Aerobic capacity | Age, maturation, body composition, training age, stature | Multilevel models |  |  |  |  |  |  |  |
| Valente-Dos-Santos et al. (2012) [42] | Repeated sprint | Age, maturation, aerobic capacity, power, body composition, training experience, stature | Multilevel models |  |  |  |  |  |  |  |
| Valente-Dos-Santos et al. (2014) [41] | Dhange of direction | Maturation, maturation, body composition, stature, aerobic capacity, power, training load | Multilevel models |  |  |  |  |  |  |  |
| Waldron et al (2014) [56] | Anthropometrics, muscular power, speed, aerobic capacity | Age | Repeated measures ANOVA |  |  |  |  |  |  |  |
| Wright & Atkinson (2019) [50] | Speed, muscular power, repeated sprint | Time | Within and between participant magnitude-based inferences |  |  |  |  |  |  |  |
| Zhao et al (2020) [57] | Anthropometrics, aerobic capacity, muscular strength | Time | Generalised linear model and repeated measures ANOVA |  |  |  |  |  |  |  |
